# Screening for anxiety and depression in active military personnel with asthma

**DOI:** 10.1192/j.eurpsy.2023.1596

**Published:** 2023-07-19

**Authors:** H. Ziedi, I. Mejri, M. Kacem, S. Mhamdi, S. Daboussi, C. Aichaouia, Z. Moatemri

**Affiliations:** 1 occupational health department, Charles Nicolle Hospital; 2 pneumology department, Tunis military hospital, Tunis, Tunisia

## Abstract

**Introduction:**

Asthma is the most common allergic respiratory disease and is frequently associated with symptoms of depression and anxiety. However, this association remains poorly understood, especially in active military personnel with asthma who are particularly exposed to a high mental and physical load.

**Objectives:**

To investigate the presence of anxiety and depression in active military personnel with allergic asthma.

**Methods:**

This is a descriptive cross-sectional study conducted at the pneumology department of the Tunis military hospital that interested active military personnel followed for asthma and who consulted the department in the period from January 1, 2022 to October 31, 2022. The Hospital Anxiety and Depression Scale (HADS) was used to assess depression and anxiety.

**Results:**

During the study period, 36 asthma patients were included. The mean age was 35±8 years with a male predominance of 83%. The majority of the participants were non-commissioned officers (91%), whom were in field positions in 68% of cases. The median professional seniority was 9 [6; 17] years. Active smoking was noted in 47% of the participants. Half of the patients had comorbidities. A history of major depressive syndrome was reported by 8% of patients. Current antidepressant treatment was mentioned by only one patient. Asthma was well controlled in 66.7% of cases. Definite anxiety was found in 30% of the patients while it was doubtful in 26% of the population. Depression was present in 18% of the participants. Specialist psychiatric care was recommended for patients with depression and anxiety.

**Conclusions:**

Anxiety and depression are significant comorbidities in asthma patients. Screening for these risks is necessary, especially in the military population whose work requires mental and physical integrity.

**Disclosure of Interest:**

None Declared

